# The impact of early visual cortex transcranial magnetic stimulation on visual working memory precision and guess rate

**DOI:** 10.1371/journal.pone.0175230

**Published:** 2017-04-06

**Authors:** Rosanne L. Rademaker, Vincent G. van de Ven, Frank Tong, Alexander T. Sack

**Affiliations:** 1 Psychology Department, University of California San Diego, San Diego, California, United States of America; 2 Cognitive Neuroscience Department, Maastricht University, Maastricht, The Netherlands; 3 Psychology Department, Vanderbilt University, Nashville, Tennessee, United States of America; University Medical Center Goettingen, GERMANY

## Abstract

Neuroimaging studies have demonstrated that activity patterns in early visual areas predict stimulus properties actively maintained in visual working memory. Yet, the mechanisms by which such information is represented remain largely unknown. In this study, observers remembered the orientations of 4 briefly presented gratings, one in each quadrant of the visual field. A 10Hz Transcranial Magnetic Stimulation (TMS) triplet was applied directly at stimulus offset, or midway through a 2-second delay, targeting early visual cortex corresponding retinotopically to a sample item in the lower hemifield. Memory for one of the four gratings was probed at random, and participants reported this orientation via method of adjustment. Recall errors were smaller when the visual field location targeted by TMS overlapped with that of the cued memory item, compared to errors for stimuli probed diagonally to TMS. This implied topographic storage of orientation information, and a memory-enhancing effect at the targeted location. Furthermore, early pulses impaired performance at all four locations, compared to late pulses. Next, response errors were fit empirically using a mixture model to characterize memory precision and guess rates. Memory was more precise for items proximal to the pulse location, irrespective of pulse timing. Guesses were more probable with early TMS pulses, regardless of stimulus location. Thus, while TMS administered at the offset of the stimulus array might disrupt early-phase consolidation in a non-topographic manner, TMS also boosts the precise representation of an item at its targeted retinotopic location, possibly by increasing attentional resources or by injecting a beneficial amount of noise.

## Introduction

Humans sense the world in a highly visual fashion–the flow of information from the eyes gives rise to an ostensibly effortless and seamless picture of our external environment. Despite its apparent simplicity, visual perception requires the brain to form an ongoing internal representation of all the information we are perceiving and perceived just moments ago, even if this information can no longer be sensed directly. Working memory takes center stage in the process of cognition by allowing relevant information to be kept online for further computation, serving as an indispensible buffer for human thought. Here, we investigated working memory for visual information and the role of early visual cortex during the maintenance of such information.

How might the brain meet the computational demands associated with working memory maintenance? The act of keeping visual memories online involves a network of frontal [1,2] and parietal [3–6] regions, as well as visual areas that were involved when the information was originally sensed [7–10]. The coordinated effort of higher-level and sensory brain regions during the short-term retention of visual information is believed to be flexible and goal dependent [11]. One dominant theory is that higher-level areas recruit sensory areas that are specialized in processing the sensory analogs of specific mnemonic contents [12–16].

It has been suggested that sensory recruitment during visual memory is achieved in a spatially global and non-retinotopic manner. For example, while people remembered an orientation presented in the left visual field, this orientation was decodable from patterns of functional Magnetic Resonance Imaging (fMRI) activity originating from *both* ipsi- and contralateral primary visual cortex (V1) [1,17]. However, the task employed did not require subjects to maintain the relevant feature (here: orientation) bound to any specific location on the screen. Therefore, the lack of retinotopic recruitment could also be interpreted as a spread of feature-based attention [18–20]. Conversely, memory for visual information does depend on retinotopically specific representations when the explicit binding of stimulus features to a particular location is required to perform a task. For example, location matters when people remember objects in a scene [21,22], when two orientations are presented one in each hemifield [9], or when location is made salient by a spatial transformation during memory [23,24].

To directly probe the causal role of sensory areas during the retention of visual stimuli, as well as the spatial extent of such recruitment, brain processing during memory can be actively altered by means of Transcranial Magnetic Stimulation (TMS). Previous work with TMS has provided support for both the necessity of visual sensory recruitment [25], as well as retinotopically specific maintenance [26,27]. However, previous TMS studies demonstrating the spatial extent of memory representations confined in a retinotopic manner suffered some drawbacks: Specificity was only found very early during retention, probably during encoding [26], or was measured indirectly via the qualitative judgment of phosphenes [27]. Thus, while these studies suggest that brain stimulation has the potential to impact visual memory performance when applied at the level of sensory representations, it remains to be seen whether retinotopically specific effects on performance can be found when TMS is applied outside of the range of sensory encoding.

While sensory recruitment during visual working memory has been well documented in studies measuring blood oxygenation with fMRI, the functional role of such recruitment is much less understood. In addition to the issue of retinotopic specificity, a second unanswered question concerns the functional role of sensory recruitment during working memory maintenance. One hypothesis is that representations in sensory cortex might be epiphenomenal [6]. However, given that sensory areas can represent information with a degree of precision not easily achieved by less specialized areas, another hypothesis is that their role during memory is to maintain high-precision representations [28].

Here, questions of *specificity* and *functional relevance* were addressed by applying TMS over occipital cortex while participants were remembering four oriented gratings, presented one in each quadrant of the visual field. By cuing memory based on spatial location, this task encouraged participants to encode and retain orientation information at the spatial locations at which they were presented (i.e. binding object identity to spatial position [29]). This design allowed us to test for retinotopically specific recruitment of visual sensory cortex during visual working memory. To probe the functional role of sensory areas during the maintenance of orientation information, we combined TMS with rigorous psychophysical testing using the method of adjustment. This procedure involved collecting many trials per participant, calculating the angular deviation between the reported and true orientation on each trial, and analyzing the resultant error distributions by fitting a mixture model [30]. A mixture model characterizes memory recall errors as having two underlying sources: response variability and the probability of random responses. We applied this model to evaluate, respectively, the effects of TMS on memory precision and the effects of TMS on the likelihood of successful memory maintenance (i.e. “guess-rate”).

We hypothesized that TMS over visual cortex would impact memory precision (but not guess-rate) in a retinotopically specific manner. There exists a clear link between information contents in visual cortex (as indexed by classification performance) and mnemonic resolution (as indexed by behavior), with more information predicting higher behavioral precision [7,31,32]. One means by which TMS could impact behavior is by locally injecting random noise. Such noise could act to reduce the amount of information at the TMS location in visual cortex, and consequently negatively impact behavioral precision. Alternatively, at low levels of noise behavioral precision could improve: If weak neural signals are below (firing) threshold, a small amount of noise can push the intensity of these weak signals above threshold, enhancing signal discriminability–an idea known as ‘stochastic resonance’. Note that effects of noise on information transfer are non-linear, because with no (or too little) noise a threshold will not be reached, while too much noise will drown out the signal. Indeed, with low-intensity TMS stimulation visual sensitivity improves [33], and behavior based on weak, but not strong, neural signals is facilitated [34]. Mnemonic signals likely rely on weak sub-threshold signals [35,36], and low-intensity visual cortex TMS during working memory has indeed been shown to benefit behavior [27,37], and can even resurrect neural representations about unattended mnemonic items [38].

Pulses were applied at two different time intervals to check for potential differences between processes occurring at the tail end of encoding, and processes occurring well within the retention phase. A previous study has shown that behavioral effects of TMS over visual cortex during working memory depends on the timing of TMS: Participants remembered a circle with two lines extending from its center, forming a wedge, and judged whether a target dot appeared inside or outside of the remembered wedge after a two-second delay. When TMS was applied at the end of the delay, responses were faster compared to vertex or no TMS stimulation. By contrast, TMS applied at the onset of the delay slowed response times [39]. Because brain stimulation interacts with ongoing neural activity at the stimulated region [40,41] we expected that TMS at different time points of the delay (encoding and maintenance), would differentially impact behavior.

Here we found that TMS applied during the short-term retention of orientation stimuli improved orientation recall in a retinotopically specific manner. This localized improvement was the result of an increase in memory precision at the TMS location. Furthermore, TMS early during retention–at the tail end of encoding–resulted in a non-retinotopic reduction in recall performance compared to TMS late during retention. This reduction in performance was the result of an increase in the likelihood of guess-like responses.

## Methods

### Participants

Eight participants were recruited from Maastricht University (5 females; mean age = 25.13 years, SE = 0.81). All had normal or corrected-to-normal vision, provided written informed consent, and passed a medical screening based on published safety guidelines [42] overseen by an independent medical supervisor. The medical ethics committee of the Maastricht University Medical Centre approved the study. With exception of one of the authors, participants received monetary reimbursement.

### Overall study design

**Fig 1:**
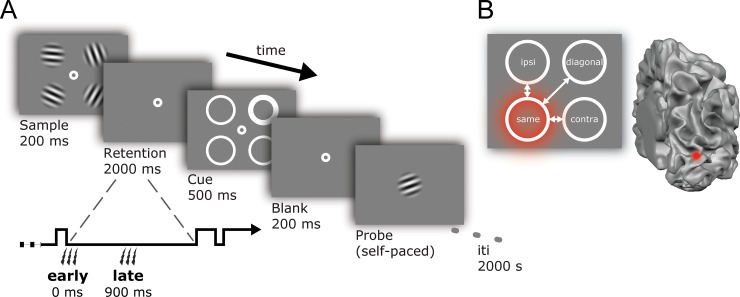
Trial sequence and relative locations. **(A)** Participants viewed a sample array with 4 randomly chosen orientations, and remembered these over a two-second delay. During the delay participants received 3 pulses of real or sham TMS over their left or right hemisphere. The pulses happened either directly at the offset of the sample array, or midway during the delay. A cue array indicated which of the four orientations was probed for recall, and after a short blank, participants rotated a test grating via button presses to match the cued orientation. **(B)** Responses at the four visual field locations were analyzed according to their position relative to the pulse. The visual field position targeted by the pulse could overlap with the memory item that was cued (‘same’), the cued item could be contralateral to the affected visual field location (‘contra’), it could be ipsilateral to it (‘ipsi’), or diagonal to it (‘diagonal’). In the example depicted here, right early visual cortex is stimulated, targeting the lower-left visual field. Consequently, the upper left position becomes ‘ipsi’, the upper right position ‘diagonal’, the lower left ‘same’, and the lower right position ‘contra’–relative to the visual field location affected by the TMS pulse.

We combined functional and anatomical MRI with neuro-navigated TMS during a psychophysical working memory task (Fig 1A). Neuroimaging was utilized for the purpose of neuro-navigation [43], allowing stable TMS stimulation sites across multiple psychophysical sessions. During the first (fMRI) session, anatomical and functional localizer data were obtained. The second (TMS) session determined the exact TMS target points (based on individually localized visual cortical activity; S1 Fig; S1 Movie) and TMS intensities to be used throughout the experiment. Participants also practiced 160 trials of the working memory task.

During the next 5–6 sessions, psychophysical data was collected while applying TMS over visual cortex (Fig 1B). A TMS coil (real or sham) was placed over either the left or the right dorsal part of early visual cortex (V1/V2). Sham TMS was used to control for attentional biasing effects that can arise from “clicking” sounds at different points in time [44]. For half of our participants, a single session consisted of 4 blocks of 80 working memory trials per block, of which three blocks involved triple-pulse TMS stimulation at 10 Hz, and one block involved triple-pulse sham stimulation at 10 Hz. Target hemisphere (left or right) and type of stimulation (real or sham) were counterbalanced over blocks, sessions, and participants. The other half of participants underwent the same procedure, with one exception: they performed the blocks of sham-stimulation separately, several months after completing the real TMS sessions.

### MRI measurements

Scanning was performed at the Maastricht Brain Imaging Center (M-BIC) on a 3.0-Tesla Siemens MAGNETOM Allegra scanner using a standard birdcage head coil. A high-resolution 3D anatomical T1-weighted scan was acquired from each participant (FOV 256 x 256, 1 x 1 x 1 mm^3^ resolution, 192 slices, MPRAGE). To measure BOLD contrast, standard gradient-echo echoplanar T2*-weighted imaging was used to collect 28 slices covering the entire occipital lobe. Scan parameters for six participants were: TR, 2000 ms; TE, 30 ms; flip angle, 80°; FOV 192 x 192; slice thickness, 3 mm (no gap); in-plane resolution, 3 x 3 mm^2^. For two other participants the scan parameters were: TR, 2000 ms; TE, 30 ms; flip angle, 90°; FOV 256 x 256; slice thickness, 2 mm (no gap); in-plane resolution, 2 x 2 mm^2^.

Preprocessing and analysis of the anatomical and functional MRI data were performed using BrainVoyager QX software (version 2.3.0.1750, Brain Innovation, Maastricht, the Netherlands). All anatomical data underwent inhomogeneity correction of signal intensity across space, and a tissue contrast enhancement using a sigma filter (7 cycles, range 5). Automatic grey-white matter segmentation was performed, after which manual corrections were made to improve segmentation over occipital cortex. The borders of the two resulting segmented sub-volumes were tessellated to produce surface reconstructions (folded meshes)–one for each hemisphere. These reconstructions were created to recover the exact spatial structure of the cortical sheet and to improve visualization of anatomical gyrification.

In the scanner, stimuli were generated using MATLAB 7.10.0 (R2010a) and the Psychophysics Toolbox [45]. Stimuli consisted of 5 Hz flickering black-and-white checkerboards (1° radius) presented 4° from fixation in either the lower left or lower right (randomly interleaved) quadrant of the screen against a uniform grey background (55.86 cd/m^2^). Stimulus locations encompassed the same visual field position as the two lower Gabor patches in the working memory task (Fig 1A). Stimuli were viewed through a mirror system on a back-projected screen (1024 x 768 resolution, 60 Hz refresh rate) at a distance of 66 cm in an otherwise darkened scanner room. We ran two 5-minute functional runs, during which a 12 second fixation period and a 12 second stimulus period alternated twelve times. Participants fixated a 0.5° white bull’s eye throughout while monitoring occasional dimming of the checkerboard (~5 times per block; detection rate 43.18% with SE = 0.04%).

After discarding the first 4 functional volumes, we applied automated 3D motion correction, slice timing correction (sinc), and high pass temporal filtering. No spatial or temporal smoothing was applied directly. Next, fMRI data were aligned to the within-session anatomical scan via rigid-body transformations, with all automated alignment carefully inspected and manually fine-tuned when necessary. Functional data from both runs were combined and analyzed using a general linear model (GLM; [46]). Activity for the left and right hemispheres was based on the statistical contrast between BOLD [47] elicited by visual stimulation in the lower right versus lower left visual field.

### TMS protocol

Localization of the TMS target points was achieved by co-registering the anatomical reconstruction of a participant’s head with the participant’s head in real space using stereotaxic data recorded with an ultrasound digitizer. Neuro-navigation was used to manually maneuver the TMS coil relative to a participant’s skull, while seeing in real-time their computer generated anatomical surface reconstruction with functional localizer activity superimposed. TMS target points were defined to lie within this region of activation. Specifically, each target point was chosen as posteriorly as possible within this region of activation, while still eliciting a phosphene overlapping the visual field location where stimuli would be presented (3–5° from fixation in lower left and right quadrants). Each TMS target point was marked on the cortical surface reconstruction by a digital marker and saved to guide neuro-navigation for all future sessions.

Biphasic TMS pulses were delivered by means of a figure-of-eight coil (MCB70) and a MagPro R30 stimulator (Medtronic Functional Diagnostics A/S, Skovlunde, Denmark). This setup allows for pulse strengths (defined as the rate of change in the magnetic pulse) up to 148 A/μs at 100% of stimulator output, and 52 A/μs at 35% stimulator output. Pulses were applied at 80% of phosphene threshold to ensure that participants did not perceive phosphenes during the working memory task. Phosphene thresholds were determined for the left and right hemispheres at the TMS target points, and kept constant throughout all experimental sessions. Thresholds were determined by starting stimulation at 60% of stimulator output, and using a 2-down 1-up procedure to find the intensity at which phosphenes were reported 50% of the time. Two participants did not experience phosphenes: stimulation intensity was set at the average intensity of other participants in the study, and target points were chosen at the peak-activity determined with fMRI.

Participants received 240 TMS pulses (3 pulses * 80 trials) during each run of the working memory task, and performed a total of 16 runs. The average pulse intensity used was 34.44% (SE = 0.54) of maximum stimulator output, with no significant difference between hemispheres (mean left = 35%, mean right = 33.88%, t = 1.386, p = 0.208).

TMS was applied in a counterbalanced fashion over left- or right early visual cortex for the purpose of scientific rigor and generalizability of our findings. A 3-way within-subject ANOVA (4 relative locations x 2 pulse timings x 2 stimulated hemispheres) on the absolute recall errors revealed no evidence to indicate differences in the stimulated hemisphere on performance (*F*_(1,7)_ < 0.001; *p* = 0.986). Therefore, all data presented here have been collapsed across hemisphere.

### Working memory task

Stimuli were generated with MATLAB 7.10.0 (R2010a) and the Psychophysics toolbox [45] under Windows XP and viewed in a dark room on a luminance-calibrated 19” Dell TFT monitor (1280 x 1024 resolution, 60Hz refresh rate). Communication between the experiment pc and stimulator was established using PortTalk V2.0 (Beyond Logic). Stimuli consisted of oriented gratings with a spatial frequency of 2 cycles/°, a diameter of 2°, and 20% Michelson contrast with a wide Gaussian envelope (sd = 2°) presented on a uniform grey background that shared the mean luminance of 40.23 cd/m^2^. Stimuli were presented at four fixed locations around a central fixation point at an eccentricity of 4°. Participants viewed the stimuli from 57 cm, and were instructed to maintain steady fixation, aided by a centrally presented white bull’s eye (0.5° diameter). A chinrest, forehead rest, and the tight placement of the TMS coil against the back of the head assisted in maintaining head stability.

Observers were presented with a 200 ms sample array of 4 to-be-remembered gratings (Fig 1A). Each grating had an independently chosen random orientation (1–180°), with the only constraint that simultaneously presented orientations differed by >10°. During a 2-second delay a TMS triple-pulse was applied at 10 Hz (i.e. 200 ms duration) either directly following the offset of the sample array, or midway through the retention interval (the first pulse occurring 900 ms into the interval). Next, 4 spatial cues appeared for 500 ms outlining the locations of the previously presented sample stimuli. A 0.15° wide white circle probed the location of the grating to be reported from memory. Non-target locations had 0.05° outlines. After 200 ms a test grating was presented centrally at an initially random orientation. Participants used separate buttons on a keyboard to rotate the test grating clockwise or counterclockwise to match the probed orientation.

Note that due to anatomical constraints, TMS can only be applied over the dorsal (and not ventral) part of visual cortex. Thus, TMS can only target the two lower (and not upper) of the four stimulus-locations probed during our memory task. Previous studies have relied on this anatomical feature to contrast behavioral performance between a ‘TMS quadrant’ (usually the lower left) and a ‘control quadrant’ (usually the upper right) [26,48–50]. In the current task, memory could be cued in all four visual field quadrants. Consequently, data were analyzed according their “relative location”, i.e. the relationship between (1) the visual field location targeted by the TMS pulse, and (2) the visual field location probed for recall. To illustrate, assume that on a given block the right hemisphere was stimulated with TMS, targeting the lower-left visual field (see also Fig 1B). If the target was subsequently cued in this lower-left quadrant, TMS and target stimulus occupied the “same” relative location. On trials where the target was cued in the upper-left quadrant (ipsilateral to the TMS location) the relative location was “ipsilateral”. Similarly, a target cued in the lower-right quadrant is at a “contralateral” relative location, whereas a target cued in the upper-right quadrant is at a “diagonal” relative location. The same logic is applied when the coil is moved to the left hemisphere, targeting the lower-right visual quadrant.

The *diagonal* location represents the *control quadrant*, as frequently employed in TMS studies of visual cortex. Such a control quadrant carries two major advantages over sham stimulation. One is that the diagonal location is probed randomly interleaved with trials probing other locations, ensuring that participants are in the same general state during both trial types. Second is that real TMS is applied even when participants are probed at the control quadrant, ensuring that the acoustics and tactile experience during these trials perfectly matches that of other probed locations.

### Analyses

In addition to looking at behavior by calculating absolute recall errors, we separately estimated the precision of memory for successfully remembered items and the likelihood of memory failure by adopting a mixture-model approach [30]. The underlying assumption of this model is that on some trials items are remembered with a certain degree of precision, whereas on other trials items are not available for recall resulting in random guesses. This assumption is implemented by fitting a circular Gaussian-shaped model to the distribution of recall errors (reported orientation minus target orientation) with two key parameters: One is the standard deviation (*SD*), or width of the circular portion of the distribution, assumed to reflect the precision of working memory for successfully remembered items (with better precision indicated by a smaller *SD*). Two is the relative proportion of area under the curve corresponding to a uniform distribution (*p-Uniform*), which captures the extent to which the entire distribution needed to be translated along the y-axis to account for the frequency of guess-like responses, assumed to reflect the probability of guessing. We rely on these two parameters because they provide a useful way to capture broad trends in the data, and because they may signify distinct types of errors. However, it is important to acknowledge that the mapping between these parameters and the underlying sources of error in the working memory system rely on assumptions regarding the exact nature of working memory performance, and that competing models have been proposed (e.g., [51–56]).

## Results

### Absolute error

The absolute error is a descriptive statistic measuring the overall degree of recall error when participants recall an orientation from memory via method of adjustment. The absolute error is calculated as the absolute difference (in °) between the reported and target orientation, and smaller errors indicate a more veridical memory of the target. We compared the absolute error at four relative distances from the visual field location targeted by TMS, and at two time points (early or late during the delay) at which TMS was applied (i.e. with a 4 x 2 repeated measures ANOVA).

**Fig 2:**
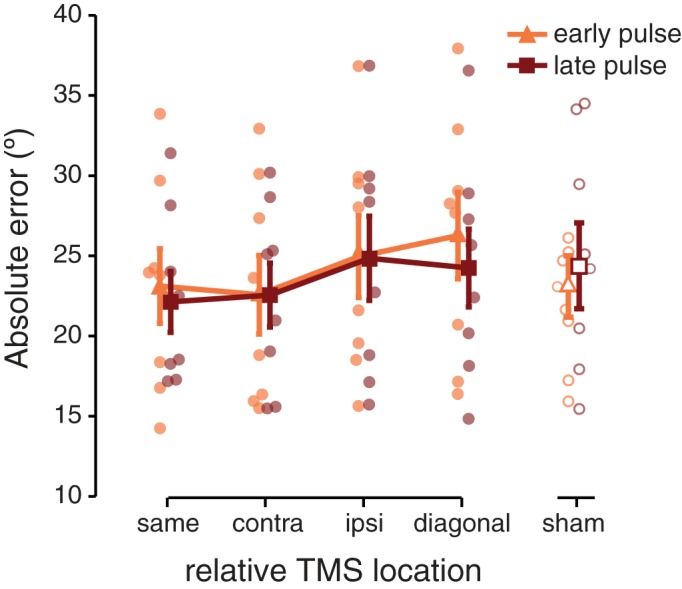
Absolute error relative the TMS location. When a memory item was cued at the same location as targeted by the TMS pulses, the absolute response error was marginally smaller than when a memory item was probed diagonally to the pulses. This indicated better performance when the memory item at the TMS location was cued (compared to the item diagonal to TMS). Early pulses resulted in marginally worse performance compared to late pulses, irrespective of the cued visual field location. Sham data (unfilled orange and red symbols) are shown collapsed across all locations, separately for early and late sham pulses. A separate statistical test showed that the time point at which audible sham clicks were delivered did not impact behavior. Transparent dots indicate individual participant data, error bars depict ± 1 SEM.

Proximity of an item maintained in memory to the visual field location targeted by TMS had a facilitative effect on memory performance, as indexed by smaller errors for items proximal to the pulsed location (main effect of relative location; *F*_(3,21)_ = 3.483; *p* = 0.034; Fig 2). Post-hoc ANOVA’s (each 2 relative locations x 2 pulse timings) were performed to investigate the origins of this main effect of location, comparing performance between all possible pairs of locations. The most prominent difference between two locations came from the comparison between trials on which the TMS pulses and target location overlapped (‘same’ condition) versus when they were furthest apart (‘diagonal’ condition, i.e. the control quadrant) (*F*_(1,7)_ = 4.721; *p* = 0.066; all other p > 0.086). Due to the nature of the study we did not correct for multiple comparisons (see also Discussion).

Comparing the two pulse-timings showed a marginally significant main effect of pulse timing (*F*_(1,7)_ = 4.820; *p* = 0.064) indicating that applying TMS pulses early, directly at the offset of the stimulus display, resulted in larger recall errors (24.23° on average) than TMS applied midway through the retention interval (23.4° on average)

Audible ‘clicks’ from a TMS machine can have differential attentional cuing effects when presented at different time points [44], and thus have the potential to influence behavior differently early versus late during a memory delay. Because sham TMS has no neural effects, we collected a total of 320 sham trials (160 per pulse timing) for each participant to investigate possible effects of pulse timing. While this is the right number of trials to probe timing effects (i.e. matching the number of trials per condition tested with real TMS), note that at each stimulus location this amounted to only 160/4 trials. This precluded the analysis of real and sham TMS data within the same statistical test, and therefore sham data were analyzed separately. We analyzed sham data by first looking at our condition of interest, pulse timing, while collapsing across all four locations. The time point at which sham pulses were applied did not significantly impact recall performance (*t*_(7)_ = 0.647; *p* = 0.538). We also compared performance for absolute location (comparing upper left, upper right, lower left, and lower right locations) with the data collapsed across pulse timing. Again, no differences were observed (*F*_(3,21)_ = 0.459; *p* = 0.714). The absence of significant differences on sham trials is important, since our manipulations could have had unintended differential attentional-cuing effects, mimicking the neural effects probed via TMS.

### Mixture-model results

To gain a deeper understanding into the functional role of early visual cortex involvement during visual memory maintenance, we fit a mixture model to each location and pulse timing condition. This allowed us to decompose the absolute errors into (1) memory precision as indexed by the mixture model *SD* (with a smaller *SD* indicating better precision), and (2) guess rates indexed by *p-Uniform* (with a larger *p-Uniform* indicating more guessing) [30].

**Fig 3:**
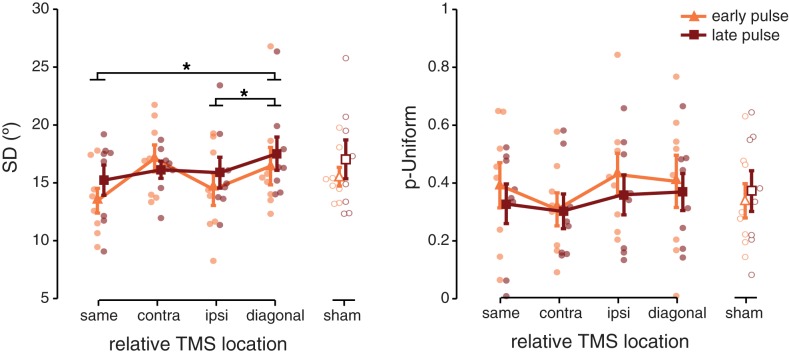
Model fits of TMS data. **(A)** Memory precision is represented by the mixed-model *SD*, with a smaller *SD* indicating more precision. Memory was most precise when the location of the target item overlapped with the location at which TMS was applied, or was ipsilateral to it, compared to pulses diagonal to the cued item. **(B)** When pulses were delivered early during the delay, participants were more likely to guess compared to pulses delivered late, irrespective of their location relative to TMS. Parameter estimates were obtained by finding the best-fitting mixture-model (centered on 0°) for the frequency distribution of each condition, using a bin width of 12° (mean R^2^ = 0.894 ± 0.029). Bin size was chosen to maximize the mean R^2^ values across experimental conditions. Transparent dots indicate individual participant data, error bars depict ± 1 SEM.

When a memory target was probed at a location proximal to the location targeted by TMS, memory recall was more precise (4 relative locations x 2 pulse timings ANOVA, main effect of relative location; *F*_(3,21)_ = 4.102; *p* = 0.019; Fig 3A). Specifically, post-hoc ANOVA’s (comparing all possible pairs of relative locations) showed that memory was more precise on trials where the pulses and target item overlapped, i.e. ‘same’ condition, (*F*_(1,7)_ = 7.974; *p* = 0.026) or were ‘ipsilateral’ to one another (*F*_(1,7)_ = 7.658; *p* = 0.028), compared to trials on which the pulses and target item were furthest apart (the ‘diagonal’ control quadrant).

No retinotopic specificity was found for the probability of uniform responses, which did not differ significantly between the four visual field locations (*F*_(1,7)_ = 1.831; *p* = 0.172). However, guess rates were higher for pulses presented early during the delay (38.42% chance on average) compared to pulses presented midway through the delay (33.97% chance on average; main effect of pulse timing; *F*_(1,7)_ = 6.594; *p* = 0.037; Fig 3B). This increase in random responses occurred irrespective of the location at which the memory item was probed (no interaction; *F*_(3,21)_ = 0.712; *p* = 0.555).

Finally, sham data were fit with a mixture model for each condition of potential interest (pulse timing, absolute location) separately, collapsing across the other condition. Timing of the sham pulses did not affect memory variability (paired-samples *t*_(7)_ = 0.822; *p* = 0.438) or guess rate (paired-samples *t*_(7)_ = 1.262; *p* = 0.247). Furthermore, no differences were found between the four absolute visual field locations for memory precision (*F*_(3,21)_ = 0.976; *p* = 0.423) or guess rate (*F*_(3,21)_ = 1.272; *p* = 0.310).

### Memory performance across the visual field

One strength of our experimental setup was that the TMS coil was fixed over the skull, which, in combination with fMRI guided neuro-navigation, ensured that the targeted brain locus remained stable relative to the four patches of retinotopic cortex excited by our stimuli. However, this setup had the obvious side effect that the stimuli were anchored onto four static visual field locations across all experimental trials. If perception or memory at these static visual field locations were anisotropic, this would complicate interpretation of our results. From the literature on basic human vision it is known that people are generally better at performing a visual task on stimuli in the lower, compared to the upper visual field. Luckily, such anisotropies are generally found for stimuli presented along the cardinal meridians, and are less prevalent (or even absent) for stimuli presented at the obliques [57,58]. Nevertheless, any residual anisotropy would impact our results by biasing performance in favor of the lower half of the visual field, incidentally, the same half of the visual field targeted with TMS.

One crucial check was to investigate how memory on sham trials compared between target stimuli in the upper left, upper right, lower left, and lower right parts of the visual field. These results were already discussed above: During sham neither the absolute error (*F*_(3,21)_ = 0.459; p = 0.714), memory precision (*F*_(3,21)_ = 0.976; *p* = 0.423), or guess rate (*F*_(3,21)_ = 1.272; *p* = 0.310) varied as a function of visual field location. While this suggests that visual field anisotropies cannot explain our findings, a null effect (absence of evidence) does not provide conclusive proof (evidence of absence). A second observation arguing against anisotropies is that, while data trends in the absolute error seem to roughly align with the idea of upper–lower visual field anisotropies (Fig 2), memory precision (Fig 3A) implies no such dichotomy.

**Fig 4:**
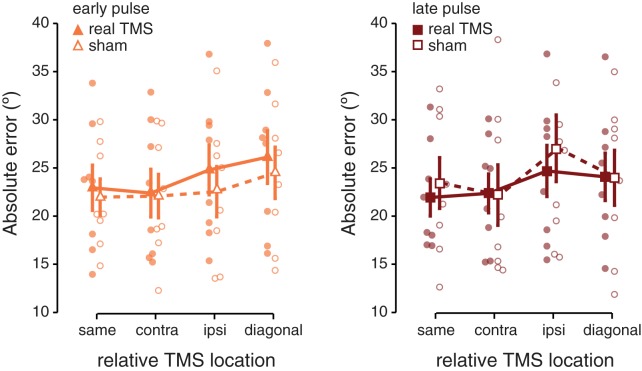
Absolute recall errors during real and sham TMS. **(A)** Performance when real or sham pulses were applied early during retention, directly at stimulus offset. **(B)** Performance for pulses (real or sham) applied midway through the retention interval. Transparent dots indicate individual participant data, error bars depict ± 1 SEM.

Finally, we wanted to directly probe whether real TMS and sham TMS interact (Fig 4), as such an interaction would provide the most convincing evidence against the idea that upper–lower visual field anisotropies (rather than TMS effects) might be driving behavioral differences at the four locations. In order to perform this comparison, we sorted the absolute errors during sham trials in a manner identical to real TMS trials: errors were determined at four “relative locations” with respect to the sham “target” location (thus, a sham coil over the right hemisphere “targeted” the lower left visual field). This resulted in an average of 160 and 40 observations per condition for real TMS and sham TMS respectively. A 3-way ANOVA comparing the 2 TMS conditions (real and sham), 2 pulse timings (early and late), and 4 locations (‘same’, ‘contra’, ‘ipsi’, and ‘diagonal’) revealed a significant interaction between pulse timing and location (*F*_(3,21)_ = 3.752; *p* = 0.027). Note that neither TMS condition and location (*F*_(3,21)_ = 0.204; *p* = 0.893) nor TMS condition and timing (*F*_(1,7)_ = 2.007; *p* = 0.2) interacted significantly.

Thus, a direct comparison of real and sham TMS did not yield conclusive results. One obvious problem is that sham data could have been more variable due to the smaller number of trials, making the comparison less robust. A more pertinent problem is that performance with real TMS is not expected to differ from performance during sham TMS at any location other than the one targeted with TMS. In fact, when we only considered visual field locations where real TMS might reasonably be expected to yield an effect (i.e. overlapping with, or ipsilateral to, the targeted location; “same” and “ipsi” in Fig 4) the interaction between TMS condition and timing did reach significance (*F*_(1,7)_ = 5.975; *p* = 0.044), suggesting that real and sham TMS differentially affect performance at different time points of memory maintenance.

## Discussion

**Fig 5:**
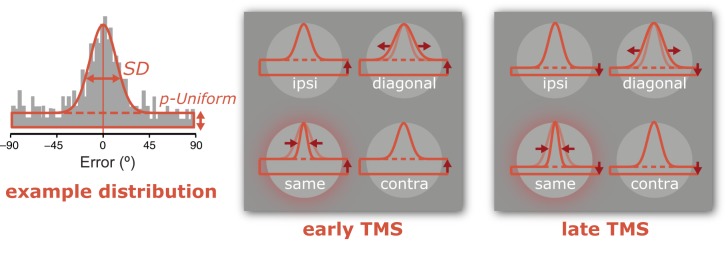
Cartoon summary of results. The left most panel depicts an example recall distribution. The frequency of recall errors (i.e. difference between response and target orientation) is indicated by the height of the grey bars. A mixture model fit of the recall errors is depicted in thick orange lines. Most responses are centered around 0°, and the precision of memory recall is captured by the mixture model *SD* parameter (smaller *SD* indicates more precision). Some proportion of responses appear to be random, indicated by the probability of guess-like responses (smaller *p-Uniform* indicates less guessing). The two rightmost panels provide a cartoon summary of our main results (exaggerated and simplified for illustrative purposes). First, the main effect of relative location is highlighted by increased memory precision (*SD*) at the location targeted by TMS (‘same’) compared to the control quadrant (‘diagonal’) for both early and late TMS pulses (compare *SD* at four visual field locations). Second, the main effect of pulse timing is shown by increased guessing when pulses were applied early compared to pulses applied late irrespective of visual field location (compare *p-Uniform* between middle and right most panels).

While participants were remembering four orientations, 10Hz triple-pulse TMS was applied over early visual cortex retinotopically corresponding to the location of one of the to-be-remembered items. Orientation recall (i.e. the absolute error) differed between the four locations at which stimuli had been presented, likely due to better performance at the location targeted by TMS compared to the location diagonal to TMS. Additionally, recall was marginally worse for early (directly at stimulus offset) compared to late (midway through retention) pulses. Recall errors were fit with a mixture model to reveal relative contributions of changes in memory variability on the one hand, and the probability of guessing responses on the other: Retinotopically specific improvements proximal to the pulse were attributed to reduced response variability, implying that memory precision can be improved locally by means of TMS. Global impairments for early compared to late pulses were due to increased guess rates, implying non-retinotopic disturbances due to TMS at the tail end of encoding. A cartoon-summary of these findings is shown in Fig 5. None of these findings were observed with sham TMS, and it is unlikely that differences in performance at the four stimulus locations due to visual field anisotropies were driving the observed TMS effects.

We would like to point out that all main effects of location were followed up with post-hoc tests that were not corrected for multiple comparisons due to the nature of our study: Use of four stimuli made our task difficult, and thus sensitive enough to observe changes in precision and guess rate both. However, our a priori hypothesis focused on comparing the “same” and “diagonal” relative locations. Thus, while exhaustively comparing all locations required 6 post-hoc tests, the majority of these comparisons were not theoretically motivated and only reported here for completeness. While the findings reported here are intriguing, this limitation renders them preliminary, and a larger study (possibly with fewer conditions) should be done to replicate these findings.

Our task encouraged binding memorized features to visual field position by making memory retrieval contingent upon spatial location. Limits on the spatial extent of sensory recruitment have been a matter of some debate, found in some cases [9] but not others [1,17]. Our finding of locally improved memory precision provides support for retinotopically specific sensory recruitment during visual memory. What is more, this local improvement was present irrespective of the time point during which TMS was applied, demonstrating that TMS can impact visual working memory beyond the sensory encoding stage.

Why might early pulses lead to higher guess rates than late pulses? Simple modulations of attention or distractibility due to the timing of the three auditory clicks emitted by the TMS coil could not explain this–using the same timing and sounds revealed no costs for early compared to late sham TMS. Guessing responses can result from forgetting, lapses of attention, or encoding failures. Since our early pulses were presented at the tail end of encoding [59–62], it is possible that TMS increased random guesses by prohibiting adequate encoding of the four stimuli. This finding is in line with previous work showing disruptions to visual memory when TMS was applied over visual cortex during the early stages of retention [26,39]. However, this earlier work observed retinotopically specific disruptions. Why might we find that early pulses affected performance across the visual field?

First, while stimulus orientations were random, accidental yet strong ensemble effects probably occurred on a portion of trials. Anticipating the fixed spatial locations of the memory stimuli, participants could have adapted a strategy relying on the constellation of the four orientations (as radial, concentric, isotropic, etc.) rather than storing features in a truly independent manner. Perceptual grouping of elements allows more of them to be stored in memory [63], and taking perceptual grouping and higher-order structures between items into account helps explain memory performance [63, 64]. Early TMS pulses applied while participants might still be extracting global shape-like representations could disrupt encoding of the whole ‘object’ through the disturbance of its local features (i.e. orientations), resulting in increased guesses for all orientations during retrieval. Such an ‘object-based’ working memory strategy might be achieved by convergent feed-forward and feedback processes at multiple stages of the visual hierarchy [65,66]–an intriguing possibility that could be tested empirically in the future.

Second, higher guess rates with early compared to late pulses imply different global cognitive ‘states’ at different stages of the delay. ‘State’ can refer to many things like attention or inattention, being trained or untrained, adapted or unadapted, etc. Specifically, it has been proposed that TMS may preferentially activate neurons in low initial activation states (i.e. low firing) relative to more active populations [41,67,68]. Alternatively, the effect of TMS on neuronal firing is monotonic, but behavioral effects (facilitation or impairment) depend on non-linearity in the input response function of the sensory neurons [33]. Either way, additional mechanisms beyond global brain state must be assumed to account for *local* improvements in memory precision that exist independent of (and in addition to) the *global* TMS timing effects reported here.

Retinotopic improvements in precision could be due to TMS enhancing processing of a memorized orientation *locally*. For example, low-intensity TMS could protect local populations of neurons at the TMS location against temporal decay by pushing weak signals above threshold. Or, participant’s attention might cycle through the four different orientations, with TMS boosting the representation of a temporarily unattended item [38]. A related idea is that TMS enhancement depends on non-monotonic intensity responses [33] as already mentioned. Here, the first basic premise would be that TMS acts via a wholesale multiplication of neural responses. The second premise would be that while remembering an orientation, the memory trace of that orientation in a population of orientation selective neurons is weak, with firing rates only slightly elevated above baseline. In terms of intensity response, the remembered orientation has a response that is slightly larger than that of not-remembered orientations. Because of the nonlinearity of intensity response profiles, a wholesale multiplication by any factor due to TMS would result in higher signal-to-noise for the remembered orientation. Conceivably, such signal-to-noise benefits could be induced with TMS on a directly perceived orientation as well, as long as contrast remains lower that the inflection point of the contrast response function, and intensive responses undergo expansive nonlinearity.

Participants in our experiment simultaneously remembered four stimuli presented at *distributed locations*, each stimulus competing for processing resources. This raises another possibility, namely that mnemonic representations interacted and that TMS yoked competition spatially in favor of the targeted retinotopic location. By enhancing neural firing at one of multiple task-relevant locations, TMS might mimic spatial attention: It’s been shown that in the absence of a visual stimulus, spontaneous firing rates in V2 ad V4 were elevated when attention was directed at a location that fell within a cell’s receptive field [69]. Thus, TMS might act as a bottom-up implementation of an otherwise top-down biasing signal, potentially by means of a spatial gating mechanism that favors the boosted location during subsequent processing stages [70].

In a more computational sense, TMS might be attenuating regulatory processes that mediate competition between orientation representations at distributed visual field locations. This can be framed in terms of a recent population-coding model of working memory [15,53,71] that assumes stimulus features (like orientation) are stored by probabilistic spiking activity in tuned populations of neurons. Critically, this model includes a broad normalization component by keeping the sum of the firing rates constant (across changes in set size or attentional prioritization, for example) [53,71]. Normalization describes neuronal responses as composed of an input ‘drive’ divided by the summed activity of a normalization pool [72,73]. Under the assumption that normalization occurs between all items in memory, TMS could be framed as increasing the input drive of neurons, biasing the overall population activity in favor of the TMS site, to the detriment of representations at non-TMS sites.

Alternatively, inhibitory interactions might exist between the four simultaneously remembered orientations. At a local level, TMS can release orientation representations from inhibitory interactions during a tilt illusion paradigm [74]. Likewise, in our study TMS could have acted to depress horizontal connections between spatially distributed representations, attenuating interference at the targeted location. In the brain, lateral connections between neurons in posterior visual areas with relatively large aggregate receptive field sizes [75], monosynaptic trans-colossal connections between the primary visual hemispheres [76], or top-down influences from for example prefrontal cortex [71,77] are all routes through which interactions between multiple and spatially distributed stimulus representations might arise.

These ‘local’ and ‘distributed’ hypotheses about improved precision at the TMS location, while thought provoking, should be tested empirically in future work to ascertain their true value. To that, we’d like to add some additional considerations regarding the work presented here. First, noisy performance from a couple of participants precluded reliable fitting using Maximum Likelihood Estimation. Instead we binned the data in bins of 12° before deriving parameter estimates. While rather coarse, this bin size was chosen to maximize R^2^ values, and results were comparable for analyses using smaller bins (i.e. 8° or 10°). Error fitting performed in this way is purely empirical, unlike model fitting, and could even be considered the better choice here. However, it should be noted that when applying a maximum likelihood approach the directionality of effects was preserved, but neither precision differences across the visual field (*F*_(3,21)_ = 1.837; *p* = 0.171), nor the pulse timing effects on guess rates (*F*_(1,7)_ = 2.854; *p* = 0.135) remained significant. Second, TMS effects are usually small, and people’s response to TMS highly variable, which is why TMS experiments generally benefit from large sample sizes. Instead, here we opted for an in depth psychophysical approach, spanning many trials and sessions. The strength of our design is that it allowed us to investigate more deeply the mechanistic underpinnings of memory maintenance and the role of visual cortex. The trade-off however, was a relatively smaller number of participants.

Despite these cautionary notes, our results provide several intriguing findings that add to an existing literature demonstrating retinotopically specific sensory recruitment [9], and dovetail previous reports of retinotopically [26] and temporally [39] specific effects of visual cortical TMS on working memory, and the notion that TMS can enhance memory representations [38]. Combining the mixture-model with TMS offered novel insights into the role of early visual cortex during the short-term retention of visual items in memory, showing a local improvement in memory precision at the TMS location, and a global increase in guess rates for TMS applied at the tail end of encoding compared to midway during the delay.

## Supporting information

S1 Bug Report Included here is the original script ran during the TMS testing sessions. A bug in this script meant the behavioral data were first reorganized before analysis, and the script doing this is also included. Finally, we have added a brief description of the bug, and where to find it in the experimental script.
(ZIP)

S1 Data Behavioral and imaging data. For each participant a.mat file is included as it was collected during the TMS testing sessions. For each participant the neuroimaging data used for targeting the TMS coil over the correct part of the brain is also included. Details about each file type can be found in the ReadMe included.
(ZIP)

S1 Fig Per hemisphere TMS target points for individual participants. Shown here are digital renderings of participant’s brains (inflated folded meshes) with localizer activity (statistical contrast between lower left versus lower right visual field stimulation) overlaid in orange/ yellow. The red circle indicates the vertex selected as target point for TMS in each hemisphere of each participant. Each target point was chosen as posteriorly as possible within the region with significant localizer activation while still eliciting a phosphene overlapping with the intended stimulus locations (in the lower left and right visual field for the right and left hemispheres respectively). For the two participants unable to perceive phosphenes (S06 and S08) the target point was positioned at the peak activity as determined via fMRI. Note that the functional activity patch of interest (or “poi”) for the right hemisphere of S02 is not shown, because we were unable to recover this data from an old laptop previously used for neuronavigation purposes in the lab (the data was of course present at the time of determining the target point).
(TIFF)

S1 Movie Localizer example. Shown is the right hemisphere of S01 with the dorsal parts of V1 and V2 depicted in dark and light pink, respectively (labels included). Next, the localizer screen as presented during scanning is shown, with a circular checkerboard in the lower left visual field. The activation induced by this localizer is overlaid on the right hemisphere in orange-yellow to show the location of the localizer activity relative to early visual regions.
(MOV)
