# Supplementary material for: The impact of early visual cortex transcranial magnetic stimulation on visual working memory precision and guess rate
Source: PLoS One. 2017 Apr 6;12(4):e0175230. doi: 10.1371/journal.pone.0175230 (PMC5383271; doi:10.1371/journal.pone.0175230)
Supplement: S1 Bug Report — A bug in this script meant the behavioral data were first reorganized before analysis, and the script doing this is also included. Finally, we have added a brief description of the bug, and where to find it in the experimental script. (ZIP) [file pone.0175230.s001.zip › S1_Bug_report/BugReport.pdf]

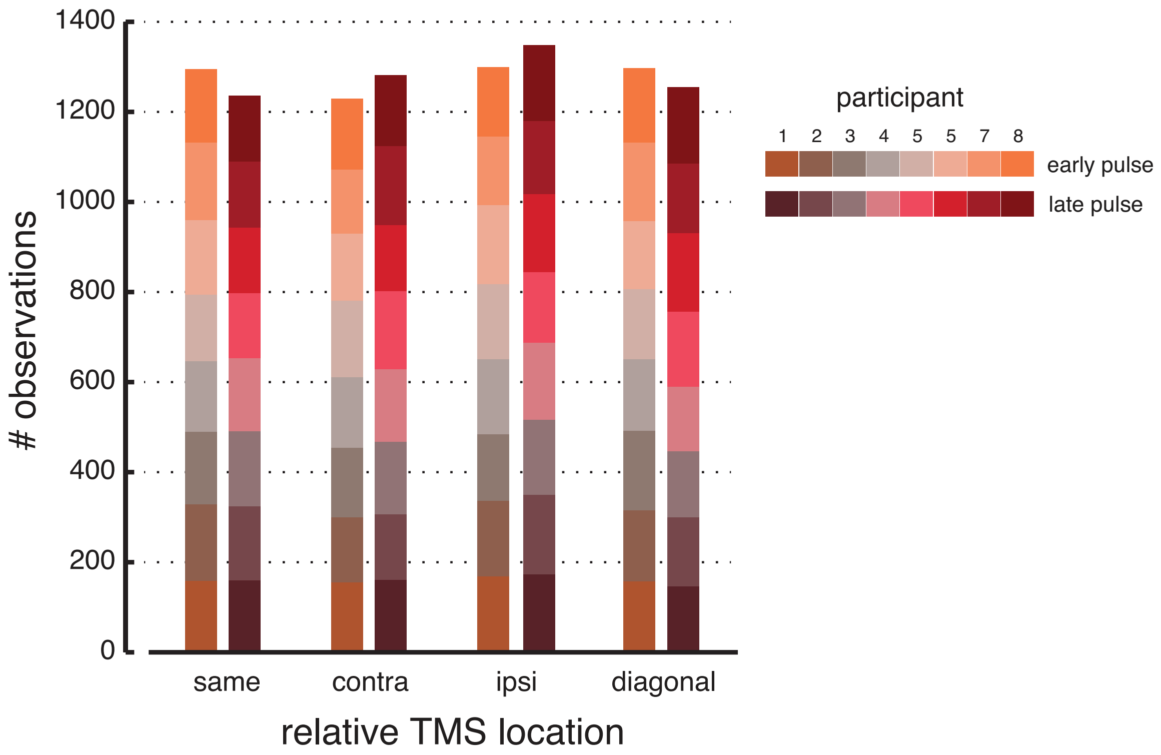

Bug report. Originally, we had meant for all conditions to be fully counterbalanced (i.e. an equal number of trials, namely 160, in each location & pulse timing condition). However, due to an unforeseen bug we ended up with a random number of trials in each condition (i.e. unequal amounts of trials, with trial numbers being reported as ~160 trials per condition). This figure shows the exact number of observations for all visual field locations and TMS pulse timing conditions across all participants. Across all participants, the smallest number of trials per condition was 1229 (early pulse, contralateral location), while the largest number of trials was 1348 (late pulse, ipsilateral location). Per participants and condition, the number of observations per condition ranged from minimally 141 and maximally 177. How this is distributed across location and pulse timing conditions is shown in the right panel. The bug can be found in our experiment script ("TMS_WM_Setsize4.m" in this folder). To understand it, first note that on lines 98–109 the to-be-presented orientations for the four orientation stimuli are defined (as "p.TargetOrients"). The orientations in the first column are designated as those that should be the target orientation. Lines 236 – 252 of the experiment script rotate all the patches around from the starting locations ("1" being the upper left position), which is supposed to make it possible to draw the orientation for (for example) the target from the first column of "p.TargetOrients", irrespective of the location of the target on the screen. Stimulus locations are saved out in lines 452–462. The bug happens when the stimulus patches are not rotated back to their starting position after every trial. Thus, on every next trial, patches are rotated as if they were in the same location as on the very first trial, which is often not the case, and stimulus locations were saved out incorrectly. The "TMS_WM_BugFix.m" script corrects for this bug, saving out the data with the correct locations.
