# Supplementary material for: The impact of early visual cortex transcranial magnetic stimulation on visual working memory precision and guess rate
Source: PLoS One. 2017 Apr 6;12(4):e0175230. doi: 10.1371/journal.pone.0175230 (PMC5383271; doi:10.1371/journal.pone.0175230)
Supplement: S1 Data — For each participant a.mat file is included as it was collected during the TMS testing sessions. For each participant the neuroimaging data used for targeting the TMS coil over the correct part of the brain is also included. Details about each file type can be found in the ReadMe included. (ZIP) [file pone.0175230.s002.zip › S1_Data/neuronavigation/ReadMe.rtf]

For each participant a number of files can be found in this folder. Here is an overview of these files + short description of their contents. These files are compatible for use with Brain Voyager software. “XX” in the filenames below is used to denote the participant number. XX_ACPC_BRAIN.vmr (or XX_TAL_BRAIN.vmr) —> Anatomical 3D image of participant’s brain (ACPC rotated, or talairach transformed). XX_ACPC_HEAD.srf —> Surface reconstruction (“mesh”) of the participants head, rotated along the ACPC axis.XX_fiducials.fdp —> Three points on the head mesh (nation, left preauricular, and right preauricular) used to coregister the Neuro Navigation digitizer coordinate system to the MRI coordinate system. XX_LH_ACPC_folded_D80.srf —> Left hemisphere surface reconstruction of folded cortex rotated along the ACPC axis. D80 indicates number of vertices. XX_LH_tgp.poi —> Left hemisphere patch of Interest indicating the TMS target point (readable by Brain Voyager, and direct translation from the XX_LH.tgp file described next).XX_LH.tgp —> Left hemisphere TMS target point (used during the actual experimental sessions, readable only by the NeuroNavigation plug-in for Brain Voyager).XX_POIs.poi —> Patches of Interest derived from the functional localizer scan. XX_RH_ACPC_folded_D80.srf —> Right hemisphere surface reconstruction of folded cortex rotated along the ACPC axis. XX_RH_tgp.poi —> Right hemisphere patch of Interest indicating the TMS target point.XX_RH.tgp —> Right hemisphere TMS target point.
